# Micronutrient Deficiencies and Anemia in Children with Inflammatory Bowel Disease

**DOI:** 10.3390/nu13010236

**Published:** 2021-01-15

**Authors:** Julie Rempel, Kanika Grover, Wael El-Matary

**Affiliations:** Section of Pediatric Gastroenterology, Winnipeg Children’s Hospital, Max Rady College of Medicine, Rady Faculty of Health Sciences, Children’s Hospital Research Institute, Winnipeg, MB R3A1S1, Canada; Julie.rempel@umanitoba.ca (J.R.); groverk@myumanitoba.ca (K.G.)

**Keywords:** anemia, child, Crohn, inflammatory bowel disease, iron, micronutrients, pediatrics, vitamin D

## Abstract

Children with inflammatory bowel disease (IBD) are at risk of developing nutrition deficiencies, particularly because of reduced intake, restrictive diets, malabsorption, and excessive nutrient loss. The aim of this study was to determine the prevalence and predictors of anemia and micronutrient deficiencies at diagnosis and one year follow up in children and adolescents with inflammatory bowel disease (IBD). Children and young adults diagnosed with IBD before the age of 17 years between 2012 and 2018 were included. Laboratory measurements including serum levels of iron, ferritin, zinc, vitamin D, vitamin A, vitamin E, selenium, copper, vitamin B12, and red blood cell (RBC) folate at diagnosis and one-year follow-up were documented as part of the Manitoba Longitudinal Pediatric Inflammatory Bowel Disease (MALPID) Cohort. A total of 165 patients with IBD were included, 87 (53%) with Crohn’s disease (CD) and 78 (47%) with ulcerative colitis (UC). The prevalence of deficiencies in our cohort at diagnosis and one year follow-up, respectively, were iron (56% and 27%), ferritin (39% and 27%), zinc (10% and 6%), vitamin D (22% and 13%), vitamin A (25% and 25%), vitamin E (5% and 4%), selenium (10 and 7%), copper (17% and 27%), vitamin B12 (2% and 5%), and Red blood cell (RBC) folate (1% and 17%). Anemia was present in 57% and 25% at diagnosis and follow up respectively. In CD patients, age of diagnosis (15y–younger than 18y) was a predictor of moderate to severe anemia and albumin levels (<33 g/L) were protective against anemia. Many children with IBD suffer from anemia and micronutrient deficiencies at diagnosis and some fail to recover after one year despite being in clinical remission.

## 1. Introduction

Crohn’s disease (CD) and ulcerative colitis (UC), collectively known as inflammatory bowel disease (IBD), are non-curable chronic inflammatory disorders of the gastrointestinal tract. Approximately 10–25% of cases begin during childhood or adolescence, and their incidence appears to be increasing [1].

Pediatric patients with active IBD are at higher risk for micronutrient deficiencies through several different mechanisms, including suboptimal oral intake, nutrient malabsorption, increased intestinal losses, systemic inflammation, hypermetabolic state, and medication’s adverse events [2,3,4]. Intestinal inflammation in pediatric IBD is associated with malabsorption, maldigestion, and gastrointestinal protein loss, contributing to deficiencies of energy, protein, and micronutrients [5]. Inflammatory mediators specifically interfere with the absorption or utilization of certain nutrients, especially iron and vitamin D [6,7].

Nutritional status has been shown to be an essential factor in determining the prognosis of IBD [8]. Micronutrient deficiencies have been shown to have significant implications on the outcomes of patients with IBD especially in those with anemia with subsequent lower quality of life and cognitive dysfunction [9]. A recent systematic review, which included 39 pediatric studies, concluded that iron and vitamin D deficiencies are common in pediatric patients with IBD, whereas vitamin B12 and folate deficiencies are rare [10]. In another study, zinc deficiency occurred at a higher rate in patients with CD than in healthy controls [11].

Anemia is a common problem in pediatric IBD patients and is reported in up to 75% of patients [12,13]. Iron deficiency anemia (IDA) is the most common form of anemia due to the lack of sufficient iron to form normal red blood cells. Iron deficiency anemia is typically caused by inadequate intake of iron, chronic blood loss, or a combination of both [14]. Serum iron alone is an unreliable marker of iron deficiency as it is influenced by a variety of factors including diurnal variation, inflammatory processes (decreased), malignancy (decreased) and menstrual blood loss (decreased). For this study, we have defined anemia using the World Health Organization (WHO) guidelines, as a decline in blood hemoglobin based on age and sex in children. Few studies that examined anemia have addressed potential predictors or factors associated with anemia such as age, sex, family history of IBD, clinical disease activity, and inflammatory biomarkers, such as high C-reactive protein (CRP), erythrocyte sedimentation rate (ESR), and albumin.

We aimed to identify serum micronutrient status at diagnosis and one-year follow-up, anemia prevalence at diagnosis and after a one-year follow-up, and factors associated with anemia at diagnosis and follow-up.

## 2. Materials and Methods

Our longitudinal, population-based cohort comprised all children and young adults (<17 years) diagnosed with IBD in the Canadian province of Manitoba between January 2011 and August 2018 and who consented to be enrolled in the Manitoba Longitudinal Pediatric Inflammatory Bowel Disease (MALPID) Registry [15,16,17]. Patients were recruited from Winnipeg Children’s Hospital, which is the only pediatric tertiary-care center in the province of Manitoba. Data on anthropometry, prescribed medications, inflammatory markers, and disease characteristics were jointly retrieved from the hospital electronic patient database and reviewed their medical and dietetic notes. All patients were diagnosed according to established clinical, endoscopic, histological, and radiological guidelines [18]. Disease location and behavior, growth impairment and perianal involvement for Crohn’s disease, and disease extent for ulcerative colitis are presented according to Paris classification [19].

### 2.1. Description of Variables

Demographic, anthropometric, clinical, laboratory, radiological, and endoscopic data were recorded for each patient at diagnosis. Height, weight, and body mass index were converted to age and sex-adjusted standard deviation scores (Z scores) using the Centre for Disease Control growth reference charts [20]. Clinical disease activity was assessed using the Physician Global Assessment (PGA) scores at diagnosis and follow up. Disease phenotype at diagnosis was categorized according to the Paris Classification [19]. Data on nutrition variables were retrieved at diagnosis (±) 7 Days, and after one year (±) 3 months. Each parameter was described both as a categorical (within normal limits or deficient) and nominal value. Anemia was defined according to age and sex normal values using WHO guidelines [21]. As per the WHO guides (hemoglobin g/L), for children 6–59 months of age; mild anemia is 100–109, moderate is 70–99 and severe is lower than 70. For children 5–11 years of age, mild anemia is 110–114, moderate is 80–109, and sever is lower than 80. For children 12–14 years of age, mild anemia is 110–119, moderate is 80–109 and severe is lower than 80. For 15 years of age and above females (non-pregnant) non-anemia is 120 or higher, mild is 110–119, moderate is 80–109 and severe is lower than 80. For 15 years of age and above males, non-anemia is 130 or higher, mild is 110–129, moderate is 80–109, severe is lower than 80. Ferritin levels were adjusted using the ECCO guidelines that recommend adjusting serum ferritin concentration by concurrently measuring C-reactive protein (CRP) to remove effects of subclinical inflammation [22].

### 2.2. Statistical Methods

The sample size calculation for this study was based on the assumption that the majority of patients would participate in the study and the proportion of patients with any micronutrient deficiency between time points or between type of disease would differ by 20%. The sample size was calculated based on a log-rank test and required at least 121 patients at each time point for each disease (CD or UC), using significance level of 5% and power of 80%. Multiple imputation by chained equations (MICE) method was used to fill in 15% of partially missing data. Twelve patients were excluded from the analysis because of incomplete initial data.

Potential discriminants of anemia incidence and the extent of its severity were defined a priori at disease diagnosis and at one-year follow-up. At diagnosis and one year follow-up these included disease type, disease phenotype in CD, extensive colitis in UC, age at diagnosis, gender, systemic biomarkers of disease activity, erythrocyte sedimentation rate (ESR), C-reactive protein (CRP), and serum albumin (AB), and family history of IBD (FH of IBD).

Continuous variables were presented either with means and SD or with medians and interquartile range (IQR) depending on the data’s distribution. Normally distributed continuous variables were described as mean and standard deviations, whereas non-normally distributed continuous variables were reported as medians and interquartile ranges (IQRs). Continuous variables were compared using simple independent *t*-tests or Mann–Whitney tests, whereas categorical variables were compared using Chi squaretests or Fisher-exact tests. Correlations between continuous variables were evaluated using Spearman p correlation or Pearson coefficients, as appropriate. Differences between groups were assessed with a 2-sample *t*-test and analysis of variance for parametric variables. Correlations between continuous variables were measured using Spearman’s rho correlation. All statistical tests were two-tailed, and *p* < 0.05 was considered statistically significant. Multivariate ordinal logistic regression was used to analyze predictors of anemia at diagnosis and one-year follow-up. For the purposes of analysis, moderate, and severe anemia types were combined, physician global assessment (PGA) moderate and severe were combined and referred to as active disease. Age of diagnosis categories included 0–younger than 12y, 12–younger than 15y, and 15–younger than 18y. For the predictive analysis at diagnosis CD and UC cohorts were modelled separately, at follow up they were modelled together. Hosmer-Lemshow test was used to calculate goodness of fit, Mcfadden pseudo R-squared was used to calculate predictive strength and the positive and negative predictive values (PPV and NPV) were calculated for anemia non-recovery at follow-up. StataCorp. 2013. Stata Statistical Software: Release 13. College Station, TX: StataCorp LPStatistical (STATA, College Station, TX, USA) was used for the analysis.

### 2.3. Ethics

The study’s protocol (HS20359 H2016:456) was approved by the Health Research Ethics Board at the University of Manitoba, Winnipeg, MB, Canada.

## 3. Results

### 3.1. Cohort Characteristics

A total of 177 children were diagnosed with IBD. 12 patients were excluded because of incomplete initial data or missing the second follow up. A total of 165 children and adolescent patients were included in the cohort: 87 (53%) with CD and 78 (47%) with UC. The median age at diagnosis was 14 (interquartile range (QR): 9.37–15.77) years. The characteristics of patients included are summarized in Table 1. Micronutrient deficiencies for all patients included at diagnosis and after one year were: iron (56% and 27%, *p* < 0.001), ferritin (39% and 27%, *p* = 0.004), vitamin D (22% and 13%, *p* = 0.013), vitamin A (26% and 25%, *p* = 0.900), vitamin E (5% and 4%, *p* = 0.565), zinc (10% and 6%, *p* = 0.162), selenium (10% and 7%, *p* = 0.258), copper (17% and 27%, *p* = 0.021), B12 (2% and 5%, *p* = 0.132), and red blood cell (RBC) folate (0.6% and 17%, *p* < 0.001). The prevalence of anemia at diagnosis was 57% (94 patients) and 25% (42 patients) at follow-up (*p* < 0.001). Only 32 of 165 patients had persistent anemia (mild, moderate, and severe) at IBD diagnosis) remained so one year after diagnosis, whereas 10 of the 72 children who were not anemic at diagnosis had anemia at one year-follow-up.

### 3.2. Crohn’s Disease

A total of 87 children (40 girls) with CD were included. The median age at diagnosis 13 (IQR: 10–15) years.

#### 3.2.1. Micronutrients

At diagnosis, prevalence of iron deficiency was 56%, falling to 23% at one-year follow-up (*p* < 0.001). Micronutrient deficiencies that improved from diagnosis to 1-year follow-up respectively included ferritin (after adjusting for acute inflammation/inflammatory markers) (21% and 15%. *p* = 00.278), zinc (14% and 3.4%, *p* = 00.012) vitamin D (16% and 6%, *p* = 00.019), vitamin A (34% and 10%, *p* < 0.001), vitamin E (6% and 2.3, *p* = 0.181), and selenium (11.5% and 9.2%, *p* = 0.640). Despite clinical improvement, the percentage of patients with subnormal copper (25.3% and 43%, *p* = 0.013), RBC folate (0% and 28%, *p* < 0.001) and vitamin B12 (1 and 3%, *p* = 0.32) increased from diagnosis to one year follow up. Clinically, there were no obvious clinical symptoms of zinc, selenium, or copper deficiency.

Serum levels of specific vitamins and minerals at diagnosis and after one year of treatment are summarized in Table 2.

We found a significant positive correlation between serum iron deficiency and copper deficiency and at diagnosis (*p* = 0.038) and with serum iron deficiency and zinc deficiency (*p* < 0.001). There was also a statistically significant positive correlation found between B12 deficiency and RBC folate deficiency (*p* = 0.0267). There were no significant associations between duodenal involvement (L4a) and anemia or RBD folate deficiency, or Ileal (L1) and B12 deficiency.

#### 3.2.2. Supplementation and Treatment (Table 3)

A total of 43 (49.0%) patients were prescribed iron supplementation at diagnosis. Of those individuals, 27 (31.0%) received oral iron compounds, 14 (16.1%) with iron salts, and two (2.3%) intravenous iron infusion. Further, 41 (47%) patients were prescribed other supplement types, which included vitamin D and a multivitamin, and 24 (27.3%) were prescribed vitamin D supplements, 11 (12.5%) were prescribed vitamin D and multivitamin (included folic acid), and six (6.8%) were prescribed a multivitamin. Supplement adherence was not captured. At diagnosis, eight (9.2%) of patients were treated with corticosteroids, 47 (54%) with exclusive enteral nutrition, 24 (28%) azathioprine (AZA), and eight (9%) with upfront anti-tumor necrosis factor (TNF) therapy. At follow-up, 38 (44%) patients were treated with azathioprine, 25 (29%) with anti-TNF medications, nine (10%) with methotrexate, and nine (10%) with combined immunomodulatory and biologic treatment.

#### 3.2.3. Anemia

The mean hemoglobin concentration at disease diagnosis was 115 ± 17 g/dL and 126.6 ± 13.41 g/dL at follow-up. Moreover, 54 (62%) patients presented with anemia at diagnosis, of which 19 (22%) were mildly anemic, and 35 (40%) patients suffered from moderate to severe anemia (Table 4). The prevalence of anemia improved from diagnosis to follow-up from 61% to 25.3% (*p* < 0.001) (Table 5).

#### 3.2.4. Inflammatory Biomarkers

At diagnosis, there was a positive association between anemia and PGA (active disease) (*p* = 0.003), and high ESR (*p* < 0.001). At one-year follow-up, there were no significant associations found.

Family History of IBD: Of the 31 (35.2%) patients who did have first-degree family history 15 (17%); 2nd degree 12 (13.6%); Other 4 (4.5%). Family history of IBD was not a significant predictor of moderate to severe anemia at diagnosis, OR = 0.34 (95%CI 0.61,1.92) or follow-up OR = 0.40 (95%CI 0.08,9.65).

#### 3.2.5. Predictors of Anemia (Moderate to Severe) at Diagnosis

35 (40%) patients had moderate to severe anemia at diagnosis. The only significant predictor of anemia at diagnosis was age of diagnosis (15 < 18). Albumin levels (<33 g/L) were protective from anemia (*p* = 0.02) (Table 6).

#### 3.2.6. Anemia at Follow-Up

Briefly, 21 (24%) patients were anemic at one year follow-up. Further, 17 patients (20%) who were anemic (mild, moderate and severe) at IBD diagnosis (n = 87) remained so one year after diagnosis, whereas five of the 33 children who were not anemic at diagnosis had anemia at one-year-follow-up. Of those who did not recover at follow up, 10 (11%) patients had mild anemia, and seven (8%) moderate to severe anemia, with a median age of 3 (IQR: 2–4) years. Moreover, 15 (17%) patients were in remission, and two (2%) had active disease. There were no significant predictors for patients who did not recover from anemia at follow-up of (Table 7).

### 3.3. Ulcerative Colitis

A total of 78 children (40 females) with UC were included. The median age at diagnosis 14 years (IQR: 11–15). Serum levels of specific trace elements, minerals, and vitamins at diagnosis and after one year of treatment are presented in Table 1.

#### 3.3.1. Micronutrients

At diagnosis and follow-up respectively, prevalence of iron deficiency was 42% and 24% (*p* = 0.001), low ferritin (after adjusting for inflammation) was 60% and 41% (*p* = 0.003), vitamin D was 28% and 21% (*p* = 0.203), vitamin E was 4% and 5% (*p* = 0.708), copper was 8% and 9% (*p* = 0.765), and zinc was 6% and 9% (*p* = 0.567). There was an increase in deficiencies of RBC folate (1% and 5%, *p* = 0.181) and vitamin A (17% and 42%, *p* < 0.001) deficiencies from diagnosis to 1 year follow-up despite clinical improvement. There were no obvious clinical symptoms of zinc, selenium or copper deficiency.

There was a significant positive correlation between copper deficiency and serum iron deficiency at follow up (*p* = 0.017). The status of serum micronutrients was not significantly associated with clinical disease activity at diagnosis or follow-up, with the exception of UC patients with zinc deficiency and active disease at diagnosis (*p* = 0.039). There was no association between 5-ASA treatment and RBC Folate deficiency.

#### 3.3.2. Supplementation and Treatment

40 (52%) patients were prescribed iron supplementation at diagnosis, 27 (34.6%) patients with oral iron compounds (type not specified in charts), iron salts 11 (14.1%), and 2 (2.6%) intravenous. A total of 41 patients were prescribed other supplements, which included 27 (34.6%) vitamin D, 10 (12.8%) vitamin D and a multivitamin (included folic acid), and three (3.8%) only multivitamins. At diagnosis, seven (9%) patients were treated with corticosteroids, 51 (65%) with 5-aminosalicylic acid (5-ASA), and three (4%) thiopurines only. During the first year of follow-up, patients received 51 (65%) with 5-ASA, nine (12%) thiopurines only, 15 (19%) were treated with 5-ASA/thiopurines combo, and three (4%) with anti-TNF medications.

#### 3.3.3. Anemia

The mean hemoglobin concentration at disease diagnosis was 114.7 ± 21.9 g/dL and 126 ± 15.5 g/dL at follow-up. The prevalence of anemia decreased from diagnosis to one-year follow-up from 52% to 27.0% (*p* < 0.001). At diagnosis, 15 (19.2%) were mildly anemia, and 26 (33%) suffered from moderate to severe anemia. Anemia type by age of diagnosis, and sex is summarized in Table 5.

Family History of IBD: Of the 36 (46%) patients who did have a family history, 14 (39%) were first degree, 13 (36%) were second degree, and 10 (29%) were others. Family history of IBD was not a significant predictor of anemia at diagnosis or one-year follow-up.

#### 3.3.4. Inflammatory Biomarkers

Positive correlations were found with anemia and active disease (PGA), CRP (>5 mg/L) and ESR (>10 mm/h) at diagnosis and follow-up. There was a significant association between anemia and ESR (>10 mm/h) at diagnosis (*p* = 0.012) and but not at follow-up (*p* = 0.38).

#### 3.3.5. Predictors of Anemia (Moderate to Severe) at Diagnosis

25 (32%) patients had moderate to severe anemia at diagnosis. There were no significant predictors of anemia for the UC cohort. Variables examined included age of diagnosis (+15y), OR = 4.6 (95%CI 0.81, 27.1), sex (female) OR = 1.99 (95%CI 0.66, 6.03), CRP (>5 mg/L) OR = 1.04 (95%CI 0.96, 1.11), ESR (>10 mm/h) OR = 1.02 (95%CI 0.99, 1.06), and albumin (<33 g/L) OR = 0.99 (95%CI 0.96, 1.01) (Table 6).

#### 3.3.6. Anemia at Follow-Up

Briefly, 21 (27%) patients were anemic at follow-up. Further, 15 (19%) patients who were anemic (mild, moderate and severe) at IBD diagnosis (n = 78) remained so one year after diagnosis. Five of the 38 children who were not anemic at diagnosis were anemic at follow-up. Of those who did not recover at follow up, seven (9%) patients had mild anemia, and eight (10%) moderate to severe anemia. Moreover, 13 (17%) patients were in remission, and two (3%) had active disease. There were no significant predictors of anemia non-recovery (Table 7).

## 4. Discussion

We found that both CD and UC patients had multiple nutritional deficiencies at diagnosis with the majority of have improved at follow-up. Over half of CD and UC patients had anemia at diagnosis with moderate improvement at one-year follow up. At diagnosis, there was a significant association between PGA and anemia, but not at follow-up. The status of serum micronutrients was not significantly associated with clinical disease activity at diagnosis or follow-up, with the exception of serum iron levels and vitamin A at diagnosis for the entire cohort (CD and UC), and for UC patients with zinc deficiency and active disease at diagnosis. However, some deficiencies in micronutrients were present even in states of clinical remission or quiescent disease at follow-up. Time to diagnosis is potentially an important factor for the development of anemia and micronutrient deficiencies. Patients who experience a longer period of symptoms prior to diagnosis could potentially be at a higher risk of deficiencies [23]. Disease activity in the proximal jejunum (as part of L4 phenotype) may also contribute to iron deficiency directly through decreased iron absorption. There were no significant correlations found between L4 and anemia in our study.

The high prevalence of serum iron deficiency correlated with rates of iron deficient anemia found in our study with both CD and UC, despite 73 (44%) CD and UC patients having received an iron supplement at diagnosis. In some studies, including ours, iron deficiency anemia is common in patients with IBD, even under complete remission, and iron sufficiency was not sustained following iron treatment due to ongoing inflammatory activity [11,24]. ECCO guidelines state the use of intravenous iron is the recommended route for moderate and severe disease, while oral iron has limited benefits [22]. In our study, the type of iron supplementation was not always included in patient notes. This may have led to an increase of reported oral iron supplementation.

Anemia is prevalent in this cohort of children with IBD, with 64% at presentation, falling to 25% at one year. At diagnosis, it is proportionally higher in those with CD versus UC (61% versus 52%). The prevalence or severity of anemia was similar among the types of IBD at diagnosis (moderate to severe anemia: CD versus UC, 40% versus 33%); mild anemia: CD vs. UC, 21% vs. 19%. Proportionally, more girls than boys were moderate to severely anemic (boys vs. girls: 15% vs. 21%; and more boys than girls had mild anemia: boys vs. girls, 23% vs. 7%). Age of diagnosis (15 < 18) and albumin levels (<33 g/L) were predictors of moderate and severe anemia at diagnosis, and only with the CD cohort. One reason could be that adolescents might be more private about their IBD symptoms, which could delay diagnosis and increase risk for anemia.

In our study, vitamin D levels followed previous studies of patients with CD and UC who had similar rates or underwent minimal improvement from diagnosis to follow-up [25,26,27]. This suggests that vitamin D status should be addressed with a more aggressive therapeutic approach. One study found that vitamin D deficiency was more common in newly diagnosed patients [25], which was also what our study found. Recent studies have investigated the relationship between vitamin D deficiency and disease severity in children with IBD, while some limited data suggested an association of vitamin D deficiency with a more severe course of disease, other studies did not report such a relationship [27,28,29]. Our study showed no statistically significant associations between vitamin D deficiency and active disease at diagnosis or follow-up. Dosage of vitamin D was not consistently captured in our cohort and thus was omitted. Additionally, patients’ low compliance with vitamin D supplementation intake may be attributed to vitamin D deficiency rates during follow-up. Two pediatric IBD randomized controlled studies did not find significant differences in the effect of different oral doses of Vitamin D ranging from 400 IU to 2000 IU [26,30], whereas one RCT did show benefit for higher range doses (either 2000 IU daily or 50,000 IU) weekly [31]. The Winnipeg IBD clinic generally prescribes 1000 IU daily. However, this information was not consistently captured in the patient charts and not included in the data.

Three studies have found low serum vitamin A levels in children with IBD, consistent with our findings. One study found that 16% of patients had low serum levels [32] and another study found that 14% of children with IBD had low serum vitamin A [33]. Another study found that 40% of patients with active CD had low serum vitamin A compared with <5% of patients with inactive CD [34]. For vitamin E, our results are consistent with two existing studies. One study reported that 6% of patients with IBD had low serum vitamin E [33], while another reported 70% with active disease, 15% with inactive disease, and 5% of controls with low vitamin E [34]. Active disease was not associated with vitamin A or vitamin E deficiencies.

Low vitamin B12 levels were rare in our study at diagnosis and at follow up. However, vitamin B12 serum levels are not always an accurate assessment of B12 as serum B12 can be false normal or false high values, even if a deficiency is present [35]. One study found 22.2% of patients with CD and 7.5% of patients with UC had low vitamin B12 status and found no difference in serum B12 concentration [36]. They also reported that patients with ileal or ileocecal resection were more likely to have abnormal serum B12 concentration; this information was not included in our study. Disease location can play a role in B12 absorption due to increased inflammation in the Ileal (L1) phenotype [37]. Energy intake is linked to disease localization in CD patients, with a reduction of energy intake only in ileal (L1) and ileocolonic (L2) disease [38]. We examined CD patients with Ileal disease and B12 deficiencies at diagnosis and follow-up and found no significant differences.

In our cohort there was a significant increase in RBC folate deficiency from diagnosis to follow-up. For CD patients, it was particular high increasing from 0–24% from diagnosis to one year follow-up. Several reasons may contribute to folate deficiency. Upon diagnosis, some IBD patients may switch to a gluten-free diet, which could increase risk for RBC Folate and B12 deficiency [39]. Some medication interactions, including 5-ASA and methotrexate, reduce cellular uptake of folate [40]. In our UC cohort, there was no association between 5-ASA treatment and RBC Folate deficiency. However, there was a statistically significant positive correlation found between B12 deficiency and RBC folate deficiency in our UC cohort, but not in the CD cohort. For the CD cohort, there was no significant association between L4 phenotype, RBC folate deficiency or anemia.

The prevalence of zinc deficiency found in our study at diagnosis was 14% in CD and 6% in UC, which is lower than reported in previous studies. Other smaller cohorts found that zinc was deficient in up to 20% of patients, mainly in those with CD [7,32,34]. Several factors can contribute to zinc deficiency in IBD, including low oral intake or poor absorption well as inflammation can be a catalyst for elevated urinary excretion of zinc. In our study there were no significant correlations between zinc deficiency and CRP, ESR, albumin, or clinical disease activity.

The prevalence of selenium deficiency in our cohort at diagnosis and follow-up was 10% and 7%; and copper was 17% and 27%. For the CD cohort, we found a significant positive correlation between copper deficiency and serum iron deficiency at diagnosis and with zinc deficiency. For the UC cohort, there was a significant positive correlation between copper deficiency and serum iron deficiency at follow up. Copper is essential for absorbing iron from the gut [41], and when copper levels are low, the body may absorb less iron. Existing studies did not consistently find low selenium serum levels in the populations. One of these studies included a small sample size (n = 24) with normal mean selenium within normal limits for patients with IBD and controls [42]. Two small pediatric cohort studies yielded contradictory results [7,43].

The strengths of our study include the prospective cohort analysis that included a relatively large number of children and adolescents at diagnosis and follow-up. The number of micronutrients examined in this study significantly add to the current limited literature There are several limitations to our study including lack of a control group and data regarding the patients’ dietary intake, time to diagnosis, and adherence to prescribed supplements (iron, vitamin D, multivitamins) and the type of supplementations. The frequency of using intravenous iron might have been under-reported in our cohort. This paper is focusing on serum levels of micronutrients and we could not find obvious clinical symptoms related to zinc, selenium or copper deficiencies. However, some symptoms like fatigue could be multifactorial and it is impossible to be certain if fatigue could be related to a specific micronutrient deficiency. Fatigue is common symptom in patients with IBD even in those in clinical remission and without anemia. Several causes of fatigue in IBD have been examined in the literature including inflammatory cytokines, nutritional deficiency, altered metabolism and psychological comorbidity. Different subtypes of fatigue could be related to different mechanisms including a possible role for bidirectional communication between the gut and central nervous system (the gut–brain axis) [44].

## 5. Conclusions

This study suggests that vitamin and mineral deficiencies and anemia are significant on-going issues in the care of children with IBD and highlights the scale of the problem even in children under follow up. Reliable diagnostic criteria are needed to help identify children who may be suitable for therapeutic intervention that, in turn, may make significant improvements in quality of life.

In general, there are limited studies in children with IBD and micronutrients, resulting in a lack of pediatric-generated medical evidence. The data could be used for screening purposes or therapeutic management. Given that early and sustained improvements in nutritional health may provide life-long advantages to children with IBD, in addition to potentially decreasing long-term healthcare costs, the medical and funding communities should recognize the importance of well-developed nutritional studies in pediatric IBD.

## Figures and Tables

**Table 1:** Cohort Characteristics.

Variable	Crohn’s Disease (n = 87)	Ulcerative Colitis (n = 78)
Age (years) of diagnosis, median (IQR)	13 (9–15)	14 (10–15)
Female, n (%)	40 (46%)	40 (51%)
Hemoglobin g/L	115 ± 16.9	115 ± 21.9
CRP ^1^ mg/dL	14.1 ± 20.0	9.3 ± 18.0
ESR ^2^ mm/L	32.6 ± 26.2	28.6 ± 25.8
Albumin g/L	35.5 ± 20.2	27.0 ± 19.7
Disease Location	Ileal (L1) 13 (15%)	Proctitis (E1) 6 (8%)
	Colonic (L2) 12 (14%)	Left-sided colitis (E2) 9 (12%)
	Ileocolonic (L3) 34 (39%)	Extensive colitis (E3) 8 (10%)
	Isolated upper disease (L4) 9 (10%)	Pancolitis (E4) 54 (69%)
	Upper disease ileocolonic (L3+L4) 19 (20%)	
	Inflammatory (B1) 70 (81%)	
	Stricturing (B2) 11 (13%)	
	Penetrating (B3) 4 (5%)	
Growth Impairment ^3^	13 (14%)	8 (10%)
Perianal Disease ^3^	15 (17%)	0
Physician Global Assessment		
Mild	60 (68%)	50 (64%)
Moderate	19 (22%)	21 (27%)
Severe	8 (10%)	7 (9%)

^1^ C-Reactive Protein [CRP], and ^2^ erythrocyte sedimentation rate [ESR]; ^3^ Disease location and behavior, growth impairment and perianal involvement for Crohn’s disease and disease extent for ulcerative colitis are presented according to Paris classification.

**Table 2:** Nutrition variables presented at Continues and Categorical (Normal of Deficient) Values in Patients with Inflammatory Bowel Disease at Time of Diagnosis and 1 year of follow-up.

Crohn’s Disease	Diagnosis	One Year Follow-Up	*p*-Value Diagnosis to Follow Up ^1^
Iron, umol/L	7.76 ± 5.6	15.4 ± 8.5	<0.01 *
Deficiency	49 (56%)	20 (23%)	
Ferritin, ug/L	54.1 ± 53.2	45.0 ± 30.8	0.28
Deficiency	18 (21%)	13 (15%)	
Vitamin D, nmol/L	65.8 ± 29	81.6 ± 34.5	0.02 *
Deficiency	14 (16%)	5 (6%)	
Vitamin E, umol/L	21.8 ± 8.0	22.1 ± 9.61	0.18
Deficiency	5 (6%)	2 (2.3%)	
Vitamin A, umol/L	1.17 ± 0.58	0.9 ± 0.28	<0.01 *
Deficiency	30 (34%)	9 (10%)	
Zinc, μg/dL	10.1 ± 2.73	10.9 ± 1.96	0.01 *
Deficiency	12 (14%)	3 (3.4%)	
Selenium, μg/dL,	1.68 ± 1.08	1.66 ± 0.25	0.64
Deficiency	10 (12%)	8 (9.2%)	
Copper, umol/L	18.0 ± 6.44	16.1 ± 6.10	0.01 *
Deficiency	22 (25%)	37 (43%)	
RBC Folate ^2^, nmol/L	2450 ± 485	2224 ± 484	<0.01 *
Deficiency	0	24 (28%)	
B12, pmol/L	492 ± 245	438 ± 256	0.32
Deficiency	1 (1%)	3 (3%)	
**Ulcerative Colitis (UC)**			
Iron, umol/L	7.86 ± 5.75	13.8 ± 9.51	<0.01 *
Deficiency	43 (55%)	24 (31%)	
Ferritin, ug/L	24.8 ± 25.7	31.0 ± 23.2	<0.01 *
Deficiency	47 (60%)	32 (41%)	
Vitamin D, nmol/L	57.2 ± 27.1	73.1 ± 29.2	0.20
Deficiency	22 (28%)	16 (21%)	
Vitamin E, umol/L	23.4 ± 8.1	22.1 ± 5.9	0.71
Deficiency	3 (4%)	4 (5%)	
Vitamin A, umol/L	1.51 ± 0.70	1.58 ± 0.55	<0.01 *
Deficiency	13 (17%)	33 (42%)	
Zinc, μg/dL	12.0 ± 2.59	12.4 ± 2.61	0.57
Deficiency	5 (6%)	7 (9%)	
Selenium, μg/dL,	1.68 ± 0.24	1.68 ± 0.24	0.21
Deficiency	7 (9%)	3 (4%)	
Copper, umol/L	18.0 ± 5.95	17.1 ± 6.13	0.77
Deficiency	6 (8%)	7 (%)	
RBC Folate, nmol/L	2495 ± 502	2179 ± 686	0.18
Deficiency	1 (1%)	4 (5%)	
B12, pmol/L	523 ± 230	486 ± 233	0.26
Deficiency	2 (3%)	5 (6%)	

^1^ Paired *t*-test was run to analyze for significant differences between micronutrient levels at diagnosis and follow-up; * Correlation is significant at the 0.05 level (2-tailed). ^2^ Red Blood Cell (RBC) Folate, The nutrition panel includes iron (normal 7.0–27.0 umol/L), ferritin (20–140 ug/L, normal 20–290 ng/mL), vitamin D (normal, 50–250 nmol/L), Vitamin E (12–46 umol/L), Vitamin A (0.9–2.5 umol/L), zinc (<8 umol/L normal 70–140 μg/dL), selenium (normal 1.33–2.03 umol/L), Copper (11–22 umol/L) B12 (normal >180 pmol/L OR 138–781 pmol/L), RBC folate (normal >1475 nmol/L), folic acid (normal 7–47 nmol/L).

**Table 3:** Supplementation and Treatment.

Crohn’s Disease (n = 87)	Diagnosis	One Year Follow-Up
Treatment ^1^		
Corticosteroids	8 (9%)	0
EEN	47 (54%)	0
Thiopurine	24 (28%)	38 (44%)
Biologics	8 (9%)	25 (29%)
MTX ^2^	0	9 (10%)
Combo ^3^	0	9 (10%)
Supplementation		
Iron total (n = 43)		
Oral	27 (31%)	
Salts	14 (16%)	
Intravenous	2 (2%)	
Vitamin D	24 (28%)	
Multivitamin	6 (7%)	
Vitamin D and Multivitamin	11 (13%)	
**Ulcerative Colitis (n = 78)**		
Corticosteroids	7 (9%)	0
Animosalcicylate	51 (65%)	51 (65%)
Thiopurine	3 (4%)	9 (12%)
Animosalcicylate and Thiopurine	17 (22%)	15 (19%)
Biologics	0	3 (4%)
Combo	0	0
**Supplements**		
Iron (n = 40)		
Oral	27 (35%)	
Salts	11 (14%)	
Intravenous	2 (3%)	
Vitamin D	27 (35%)	
Multivitamin	3 (4%)	
Vitamin D & Multivitamin	10 (13%)	

^1^ At one-year follow-up 6 CD patients were not on any treatment; ^2^ Methotraxate [MTX]; ^3^ Combo includes both immunomodulatory and biologic treatment.

**Table 4:** Iron Deficiency Anemia ^1^ grouped by sex and age at Diagnosis and Follow up ^2^.

	Diagnosis ^3^		Follow Up ^4^	
	Mild Anemia	Moderate and Severe Anemia	Mild Anemia	Moderate and Severe Anemia
**Crohn’s Disease**				
Sex				
Female ^5^	5 (6%)	20 (23%)	3 (3.5%)	6 (7%)
Male ^6^	14 (16%)	15 (17%)	9 (10%)	3 (3.5%)
Age of diagnosis				
0 < 12	3 (3%)	20 (23%)	2 (2%)	3 (3.5%)
12 < 15	8 (9%)	7 (8%)	4 (5%)	3 (3.5)
15 < 18	8 (9%)	8 (9%)	6 (7%)	3 (3.5)
**Ulcerative Colitis**				
Sex				
Female ^7^	6 (8%)	15 (19%)	4 (5%)	6 (8%)
Male ^8^	9 (12%)	10 (13%)	4 (5%)	7 (9%)
0 < 12	4 (5%)	6 (8%)	1 (1%)	1 (1%)
12 < 15	6 (8%)	14 (18%)	4 (5%)	5 (6%)
15 < 18	5 (6%)	5 (6%)	5 (6%)	5 (6%)

^1^ Iron deficiency Anemia defined by WHO; ^2^ Only patients with anemia are included in table; ^3^ At diagnosis 54 CD patients and 40 UC patients had anemia; ^4^ At follow-up, 21 CD patients and 21 UC patients had anemia; ^5^ 15 girls with CD did not have anemia at diagnosis; ^6^ 18 of boys with CD did not have anemia at diagnosis; ^7^ 19 girls with UC did not have anemia at diagnosis; ^8^ 19 of boys with UC did not have anemia at diagnosis.

**Table 5:** Iron Deficiency Anemia and Micronutrient Deficiencies Grouped by Physician Global Assessment (PGA) at Diagnosis and Follow-up for CD and UC.

	PGA Diagnosis			PGA Follow Up		
	Mild	Moderate to Severe	*p*-Value ^1^	Mild	Moderate to Severe	*p*-Value ^1^
No Anemia	55 (33%)	16 (10%)	0.001 *	114 (69%)	9 (5%)	0.409
Mild Anemia	26 (16%)	8 (5%)		21 (13%)	1 (1%)	
Moderate to Severe Anemia	29 (18%)	31 (19%)		17 (10%)	3 (2%)	
Serum Iron Sufficient	57 (35%)	16 (10%)	0.006 *	113 (68%)	8 (12%)	0.316
Deficiency	53 (32%)	39 (24%)		39 (24%)	5 (3%)	
Ferritin Sufficient	66 (40%)	34 (21%)	0.822	112 (68%)	8 (5%)	0.345
Deficiency	44 (27%)	21 (13%)		40 (24%)	5 (3%)	
Vitamin D Sufficient	91 (55%)	39 (24%)	0.110	133 (81%)	11 (7%)	0.765
Deficiency	20 (12%)	16 (10%)		19 (12%)	2 (1%)	
Vitamin E Sufficient	104 (63%)	53 (32%)	0.608	146 (88%)	13 (8%)	0.466
Deficient	6 (4%)	2 (1%)		6 (4%)	0	
Vitamin A Sufficient	89 (54%)	33 (20%)	0.004 *	114 (69%)	9 (5%)	0.647
Deficient	21 (13%)	22 (13%)		38 (23%)	4 (2%)	
Zinc Sufficient	100 (61%)	48 (29%)	0.469	143 (87%)	12 (7%)	0.797
Deficient	10 (6%)	7 (4%)		9 (5%)	1 (1%)	
Selenium Sufficient	100 (61%)	48 (29%)	0.469	142 (86%)	12 (7%)	0.877
Deficient	7 (4%)	48 (29%)		10 (65)	1 (1%)	
Copper Sufficient	91 (55%)	46 (28%)	0.883	112 (68%)	9 (5%)	0.727
Deficient	19 (12%)	9 (5%)		40 (24%)	4 (2%)	
RBC folate Sufficient	109 (66%)	55 (33%)	0.216	126 (76%)	11 (7%)	0.619
Deficiency	1 (1%)	0		26 (16%)	2 (1%)	
B12 Sufficient	107 (65%)	55 (33%)	0.216	145 (88%)	12 (7%)	0.874
Deficiency	3 (2%)	0		7 (4%)	1 (1%)	

^1^ Pearson Chi squared test was run to evaluate observed differences between variables. * Correlation is significant at the 0.05 level (2-tailed).

**Table 6:** Predictors ^1^ of Anemia (moderate to severe) at Diagnosis.

	Odds Ratio	95% CI	*p*-Value
**Crohn’s Disease ^2^**			
Age of diagnosis (15 < 18)	4.92	1.17–20.8	0.03 *
Sex (Female)	1.23	0.41–3.71	0.72
PGA ^3^ (active disease)	3.78	0.53–26.6	0.18
CRP ^3^ (>5 mg/L)	1.05	0.99–1.12	0.12
ESR ^4^ (>10 mm/h)	1.01	0.98–1.04	0.68
Albumin (<33 g/L)	0.96	0.92–0.99	0.02 *
**Ulcerative Colitis ^5^**			
Age of diagnosis (15 < 18)	4.67	0.81–27.1	0.09
Sex (Females)	1.99	0.66–6.03	0.22
PGA (active disease)	0.80	0.11–5.81	0.83
CRP (>5 mg/L)	1.04	0.98–1.11	0.21
ESR (>10 mm/h)	1.02	0.99–1.06	0.16
Albumin (<33 g/L)	0.99	0.96–1.02	0.39

^1^ Multivariate Ordinal Linear regression was used to analyze predictors at follow-up; ^2^ CD cohort n = 87 Pseudo R2 = 0.080, Hosmer-Lemeshow *p* = 0.726; ^3^ Physician Global Assessment [PGA], ^4^ C-Reactive Protein [CRP], and ^4^ erythrocyte sedimentation rate [ESR]; ^5^ UC cohort n = 78 Pseudo R2 = 0.096, Hosmer-Lemeshow *p* = 0.5751. * Correlation is significant at the 0.05 level (2-tailed).

**Table 7:** Predictors ^1^ of anemia non-recovery ^2^ at 1 year follow up for CD and UC ^3.^

CD and UC (n = 32)	Odds Ratio	95% CI	*p*-Value
Age of diagnosis (12 < 15)	1.66	0.13–21.1	0.70
Sex (Females)	3.30	0.41–26.5	0.26
PGA ^4^ (active disease)	11.0	0.36–342	0.17
CRP ^5^ (>5 mg/L)	1.05	0.93–1.19	0.40
ESR ^4^ (>10 mm/h)	1.03	0.98–1.09	0.24
Albumin (<33 g/L)	0.99	0.93–1.05	0.73

^1^ Multivariate Ordinal Linear regression was used to analyze predictors at follow-up; ^2^ anemia non-recovery are patients with anemia at diagnosis that still had anemia at follow-up, includes CD and UC patients (n = 32); ^3^ anemia non-recovery CD and UC (n = 32) Pseudo R2 = 0.155; ^4^ Physician Global Assessment; ^3^ C-Reactive Protein [CRP]; ^5^ erythrocyte sedimentation rate [ESR]. PPV and NPV calculated after model predicted for predictors of anemia at follow-up (n = 32). NPV = 0.706, PPV = 0.533. A = 12 (True negative), B = 7 (False negative), C = 5 (False positive), D = 8 (True positive).

## Data Availability

The data underlying this article were provided by permission from patients and approved by the Health Research Ethics Board. Due to privacy agreements line item data cannot be shared.
